# Lipid research toward advancing plant performance and environmental sustainability

**DOI:** 10.1093/jxb/erag195

**Published:** 2026-06-09

**Authors:** Aruna Kilaru, Doug K Allen, Marna D Yandeau-Nelson, Yuki Nakamura

**Affiliations:** Department of Biological Sciences, East Tennessee State University, Johnson City, TN 37614, USA; Donald Danforth Plant Science Center, St. Louis, MO 63132, USA; Department of Genetics, Development & Cell Biology, Iowa State University, Ames, IA 50011, USA; RIKEN Center for Sustainable Resource Science (CSRS), Yokohama 230-0045, Japan; Department of Biological Sciences, Graduate School of Science, The University of Tokyo, Tokyo 113-0033

**Keywords:** Environmental adaptation, lipid droplets, lipid metabolism, lipid regulation, lipidome remodeling, plant development, plant stress responses, spatial organization, sustainable plant production

**Lipids perform a remarkable range of functions in plants, extending far beyond their classical roles as membrane constituents and storage reserves. They participate in developmental regulation, cellular organization, stress perception and response, mechanical barrier formation, carbon and energy storage, and the integration of metabolism with environmental and ecological cues. Recent advances in analytical, imaging, and computational approaches have accelerated progress in understanding how lipid diversity, spatial organization, and dynamic remodeling contribute to plant performance under changing conditions. This Special Issue brings together perspectives that span fundamental mechanisms to applied contexts, highlighting how plant lipid research is informing strategies relevant to agricultural productivity, food security, environmental resilience, and sustainable use of biological resources.**

Plant lipid research has expanded rapidly over the past decade, driven by growing recognition that lipid function cannot be understood solely through localization, composition, or abundance. Instead, attention has shifted toward regulatory complexity, spatiotemporal organization, and dynamic remodeling of lipid pools in response to developmental cues and environmental change. Advances in analytical lipidomics, metabolite imaging and visualization, and computational analysis have enabled increasingly fine-scale resolution of lipid diversity, revealing conserved patterns of lipid remodeling and function, alongside lineage- and tissue-specific organization.

Lipid biology has become increasingly integrated with broader questions in plant science, including how plants maintain cellular integrity under stress, balance growth with defense, and allocate carbon and energy efficiently across organs and life stages. These themes have been explored across multiple recent collections in the field, reflecting a convergence of interests from basic metabolism and cell biology to whole-plant stress physiology and crop improvement. The contributions in this Special Issue build on this momentum, focusing on how lipid diversity, organization, and regulation intersect with plant performance in ways relevant to improving food security, environmental resilience, and sustainable use of plant resources.

## Functional diversity and regulation of plant lipids

Plant lipids encompass an extraordinary breadth of chemical and functional diversity, enabling roles that span structural integrity, metabolic control, developmental regulation, physiological adaptation, and plant protection. Rather than acting as a uniform class of molecules, lipids participate in highly specialized processes that are shaped by biosynthetic origin, subcellular localization, and regulatory context. The papers in this section illustrate how lipid function emerges from this diversity, highlighting spatiotemporal regulatory mechanisms that operate across scales, from transcriptional networks to enzyme complexes to whole-plant traits.

Long-chain linear isoprenoids represent a striking example of lipid diversity, with essential yet often underappreciated biological roles. Gutkowska *et al.* (2026) synthesize the current understanding of plastidial and extraplastidial isoprenoid polymers, emphasizing their involvement in photosynthesis, respiration, protein glycosylation, and reproductive processes. By placing these compounds in a physiological and evolutionary context, this work underscores how seemingly specialized lipid classes can exert broad influence on plant performance and fitness, particularly through their integration into core metabolic and cellular processes.

At the level of fatty acid biosynthesis, regulation of acetyl-CoA carboxylase (ACCase), a rate-limiting step of *de novo* fatty acid synthesis, exemplifies the complexity that is increasingly recognized in lipid metabolic control. Balbuena *et al.* (2026) describe how non-catalytic effector proteins, including biotin attachment domain-containing proteins, carboxyltransferase interactors, and PII proteins, modulate heteromeric ACCase activity in plastids. These regulatory components introduce multiple layers of control over flux into *de novo* fatty acid synthesis, linking lipid production to cellular homeostasis, nutrient status, and developmental cues. Importantly, this work reframes ACCase not simply as a known rate-limiting enzyme, but as a regulatory hub whose activity reflects coordinated input from metabolism and signaling networks.

Regulation of lipid synthesis and function also extends beyond enzymatic control to transcriptional regulation that shapes lipid composition and distribution during development and in response to environmental stress. Liu *et al.* (2026) review the transcriptional control of cuticle formation, highlighting conserved transcription factors that direct lipid biosynthesis and deposition in a tissue- and stage-specific manner. Recent studies reviewed and contextualized by Liu *et al*. reveal that cuticle synthesis and cell fate are significantly intertwined, as epidermal cell differentiation and spatial patterning of stomata and trichomes, and synthesis and deposition of cuticle building blocks are co-regulated at the transcriptional level. The cuticle provides a clear example of how lipid diversity and regulation translate directly into functional outcomes, including protection against desiccation, pathogen entry, and other environmental stresses, while also mediating developmental processes such as organ separation and reproduction.

Taken together, these contributions emphasize that functional diversity in plant lipids arises not only from chemical variation, but also from complex regulatory mechanisms that integrate metabolism, development, and physiology. A recurring theme is that lipid pathways are rarely isolated; instead, they are embedded within broader networks that allow plants to fine-tune lipid composition and flux in response to internal and external demands. This perspective sets the stage for subsequent sections, where spatial organization, environmental responsiveness, and broader physiological implications of lipid regulation are explored in greater detail.

## Cellular organization and dynamics of lipid metabolism

Beyond chemical diversity and regulatory control, lipid function in plants is strongly influenced by spatial organization within cells. Specific types of lipids are unevenly distributed across membranes, organelles, and storage compartments, and this organization is dynamic, responding to developmental state and environmental conditions. Increasingly, lipid droplets have emerged as central players in this spatial regulation, functioning not only as repositories for neutral lipids but also as dynamic organelles that interface with multiple metabolic and cellular processes and influence the movement and spatial distribution of specific lipids.

Dabisch *et al.* (2026) examine the plasticity of the lipid droplet proteome across plant evolution, development, and stress responses. Their review highlights that lipid droplet-associated proteins are neither universally conserved across plants nor static; rather, they vary substantially across species, tissues, and physiological contexts. Importantly, this work emphasizes that changes in lipid droplet composition are closely linked to functional outcomes, including lipid mobilization during development, responses to abiotic stress, and coordination with other organelles. By considering lipid droplets within an evolutionary framework, the authors reinforce the idea that droplet-associated functions have been diversified alongside plant complexity, enabling flexible lipid management across life stages.

Complementing this perspective from an evolutionary and cellular standpoint, Blot *et al.* (2026) focus on the dynamic behavior of lipid droplets within cells, emphasizing remodeling, intracellular trafficking, and physical interactions with other organelles. Lipid droplets are shown to participate actively in cellular processes, forming transient contacts with peroxisomes, mitochondria, and autophagic structures that facilitate lipid exchange and metabolic integration. Rather than being isolated storage bodies within the cytoplasm, lipid droplets function as mobile hubs that connect lipid metabolism with energy balance, redox homeostasis, and cellular stress responses. This dynamic view challenges earlier static models of lipid storage and underscores the importance of spatial context in understanding lipid function.

Together, these contributions point toward a conceptual shift in how lipid metabolism is viewed at the cellular level. Lipid droplets provide a flexible platform for coordinating lipid synthesis, storage, and mobilization, allowing plants to buffer metabolic fluctuations and adjust resource allocation when conditions change. This perspective aligns with growing evidence that spatial organization is a critical determinant of metabolic efficiency and resilience, particularly under stress or during developmental transitions.

Looking ahead, a major challenge will be to integrate molecular, spatial, and temporal information to better understand how lipid organization influences plant performance. Advances in metabolite imaging and non-invasive analytical approaches, also discussed in this issue, are beginning to provide the resolution needed to link lipid composition with cellular architecture. As these tools advance, they will enable models that are more predictive of lipid behavior in living tissues, thereby linking cellular dynamics with whole-plant traits relevant to growth, adaptation, and productivity.

## Lipids in environmental responses and adaptation

Environmental variability presents continual challenges to plant growth and survival, requiring flexible physiological strategies that operate across temporal and spatial scales. Lipids play central roles in these responses, both as structural components that stabilize cellular membranes and as signaling molecules that coordinate perception and response to cellular and environmental stress. The contributions in this section highlight how lipid remodeling and lipid-mediated signaling constitute conserved, yet adaptable, mechanisms that enable plants to respond to thermal, nutritional, and biotic challenges.

Using a systematic meta-analysis of large-scale lipidomic datasets collected under heat or cold stress conditions, Sathasivam *et al.* (2026) identify conserved, as well as unique, patterns of lipidome remodeling under temperature stress across diverse plant species, tissues, and developmental stages. Despite variation in experimental systems, common trends emerge across plant species, including shifts in membrane lipid headgroup composition, changes in acyl chain unsaturation, and sequestration of polyunsaturated fatty acids into neutral lipids. These findings suggest that plants rely on a limited set of biophysical strategies to maintain membrane integrity and function under temperature extremes, providing a unifying framework for understanding thermal adaptation at the lipid level. Together with insights into underlying genetic networks, this work lays an important foundation for plant breeding and engineering approaches to enhance crop sustainability under extreme temperatures.

Complementing this broad view, Pandey *et al.* (2026) focus on lipid-mediated responses to nutrient limitation and other abiotic stresses, emphasizing the signaling roles of phospholipids, sphingolipids, oxylipins, and lipid-derived messengers. Their synthesis highlights how lipid turnover and remodeling allow plants to conserve limiting nutrients, reconfigure membrane composition, and activate downstream signaling pathways that coordinate growth, transport, and stress mitigation. Lipid droplets and lipophagy re-emerge as important components of these responses, linking stress signaling with carbon and energy management.

Beyond abiotic stress, lipid-derived molecules also mediate interactions between plants and their biotic environment. Matsui (2026) examines the biosynthesis and function of green leaf volatiles, highlighting their rapid production in response to tissue damage and their roles in plant–insect communication and defense. These volatile lipids illustrate how lipid metabolism extends beyond intracellular processes to shape ecological interactions, enabling plants to influence their immediate environment and communicate stress signals across tissues and neighboring plants and non-plant organisms.

Together, these studies emphasize that lipid-based responses to environmental challenges are neither isolated nor purely reactive. Instead, they reflect integrated strategies that combine membrane remodeling, signaling, and metabolic reallocation to maintain cellular function and organismal fitness. A recurring theme is the balance between conservation and flexibility: while core lipid responses are conserved across plant lineages, their regulation and expression are finely tuned to specific stresses, developmental contexts, and ecological settings. Understanding how these lipid-mediated strategies intersect and are regulated will be essential for predicting plant responses to increasingly variable environments.

## Methods and perspectives for lipid function and improvement

Insights into lipid regulation, organization, and environmental responsiveness increasingly shape how lipid traits are measured and modified in plant systems. Advances in plant lipid research depend on enhanced technologies to resolve lipid composition, distribution, and dynamics in living tissues, as well as to translate mechanistic insights into measurable phenotypic outcomes. As datasets increase in dimensionality, conceptual frameworks will be essential for translating lipid complexity into biological insight. The articles in this section illustrate how methodological innovation and targeted biological studies together are shaping current perspectives on lipid function and its potential manipulation for crop improvement and enhanced bioproduct formation.

To understand lipid function, it is critical to consider lipid localization within cells and across tissues. Borisjuk *et al.* (2026) review the principles and applications of lipid magnetic resonance imaging (MRI) in plant systems, highlighting the value of non-invasive, three-dimensional approaches for studying lipid distribution in intact tissues. In contrast to destructive analytical methods, MRI enables repeated measurements within the same sample and preserves spatial context, making it particularly powerful for investigating lipid accumulation and mobilization in dense organs such as seeds. Beyond technical advances, this work underscores how spatially resolved lipid information can inform questions of developmental regulation, metabolic heterogeneity, and trait variability, providing a bridge between cellular processes and whole-organ phenotypes.

At the interface of mechanism and potential improvement, Yang *et al.* (2026) examine non-specific phospholipase C enzymes and their emerging roles in lipid signaling and stress responses. By modulating membrane lipid composition and generating signaling intermediates, these enzymes influence diverse physiological processes, including growth regulation and stress adaptation. Importantly, this review highlights how perturbation of lipid turnover can have system-wide consequences, reinforcing the need to consider regulatory networks and trade-offs when targeting lipid pathways for modification.

Gautam *et al.* (2026) focus on the development of intermediate oilseed crops, such as pennycress and camelina that can be cultivated off-season on farmland used for cash crops, as platforms for producing renewable fuels and value-added bioproducts. Rather than presenting these systems as isolated case studies, the review situates crop engineering efforts within a broader understanding of lipid biosynthesis, storage, and allocation. Furthermore, a cropping system strategy is proposed that integrates intermediate oilseed crop production with biomass crops engineered for high-yield vegetative oil production. Collectively, this work emphasizes that successful enhancement of lipid traits relies on coordinated regulation of metabolism across source, sink, and storage processes, echoing themes raised throughout this Special Issue.

Taken together, these contributions highlight a growing convergence between analytical capability, mechanistic insight, and applied perspective in plant lipid research. As methods for visualizing and quantifying lipids become more sophisticated, they enable increasingly precise interrogation of lipid function in complex biological contexts. At the same time, studies aimed at modifying lipid pathways underscore the importance of grounding translational efforts in a deep understanding of lipid regulation, organization, and interaction with other cellular processes. Moving forward, progress in this area will depend not on isolated technological advances or single-pathway interventions, but on integrative approaches that connect lipid biology across scales, from molecules to tissues to whole plants.

## Closing perspective

The contributions assembled in this Special Issue reflect a field that has moved beyond describing lipid composition in plants to revealing how lipid diversity, organization, and regulation shape plant function across scales. A unifying message is that lipid pathways operate as integrated networks, linking metabolism, development, and environmental responsiveness rather than acting as isolated modules. As analytical and imaging approaches continue to improve, opportunities will expand to connect molecular detail with spatial and physiological context. Progress in plant lipid research will therefore depend on maintaining this integrative perspective, ensuring that advances in mechanisms, methodology, and applications inform one another as plants are studied and lipid traits are modified for improved performance under increasingly variable environmental conditions.
